# Does applying CPAK classification boundaries using measurements from a surgical navigation system result in different class distributions? A retrospective assessment

**DOI:** 10.1002/jeo2.70736

**Published:** 2026-06-16

**Authors:** Matthew D. Hickey, Asim Khan, Alexander Tham, Meraj Akhtar, Antony J. Hodgson, Alistair Ewen, Frederic Picard

**Affiliations:** ^1^ School of Biomedical Engineering The University of British Columbia Vancouver British Columbia Canada; ^2^ Department of Orthopaedics Golden Jubilee National University Hospital Clydebank Glasgow UK; ^3^ Department of Mechanical Engineering The University of British Columbia Vancouver British Columbia Canada

**Keywords:** coronal alignment, radiographs, surgical navigation, total knee arthroplasty

## Abstract

**Purpose:**

Coronal Plane Alignment of the Knee (CPAK) classification defines knee phenotypes using two factors: arithmetic hip‐knee‐ankle angle (aHKA) and joint line obliquity (JLO) measured via long leg radiographs (LLRs), which can produce errors due to variability in patient positioning. Navigation‐based technology has been shown to increase accuracy and may be used where LLRs are not routine. Given this, we asked: (1) Does applying the CPAK classification system to measurements obtained from a surgical navigation system result in different class distributions when compared to LLRs? and (2) Given a mechanical alignment approach, what is the accuracy of a navigation‐based system in delivering the targeted postoperative CPAK Type V classification?

**Methods:**

We analysed data from 4087 total knee arthroplasty (TKA) procedures undertaken using surgical navigation at our institution between March 2007 and October 2022. Of these patients, 80 had preoperative LLRs from which medial proximal tibial ankle (MPTA) and lateral distal femoral angle (LDFA) were measured to calculate aHKA and JLO. We compared the mean preoperative and postoperative distributions of these alignment variables and classified patients into CPAK types and compared CPAK class frequencies between pre and postoperative TKA.

**Results:**

The mean signed differences between preoperative MPTA and LDFA values measured using surgical navigation and LLRs were 0.2° (*σ* = 2.8°, *p* = 0.46) and 1.4° (*σ* = 2.6°, *p *< 0.01), respectively. The mean signed differences in preoperative aHKA and JLO were calculated to be −1.7° (*σ* = 3.7°, *p *< 0.01) and 1.2° (*σ* = 3.8°, *p *< 0.01). Postoperatively, 87.3% of cases were classified as Type V.

**Conclusions:**

While surgical navigation yields population‐level CPAK distributions similar to LLRs, individual class assignment was preserved in only 38.8% of patients showing that measurement modality frequently alters phenotype classification. Discrepancies likely arise from differing angle definitions, cartilage assessment techniques and the non‐weight‐bearing nature of navigation compared to standing radiographs.

**Level of Evidence:**

Level III.

**Abbreviations**
aHKA: arithmetic hip‐knee‐ankle angle
CPAK: coronal plane alignment of the knee
JLO: joint line obliquity
LDFA: lateral distal femoral angle
LLR: long leg radiograph
mHKA: mechanical hip‐knee‐ankle angle
MPTA: medial proximal tibial ankle

## INTRODUCTION

**Figure 1:**
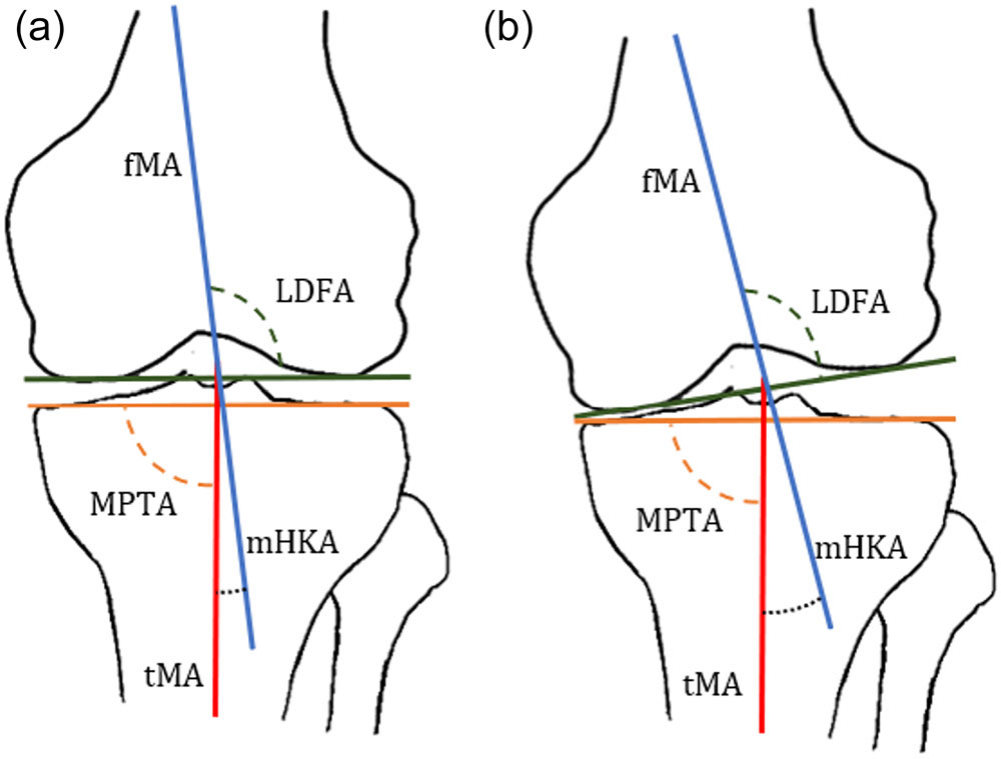
Statement depiction of the geometry of the left lower limb showing the lateral distal femoral angle (LDFA) measured relative to the femoral mechanical axis (fMA) and the medial proximal tibial angle (MPTA) measured relative to the tibial mechanical axis (tMA), as well as the overall mechanical hip‐knee‐ankle angle (mHKA) in (a) a knee with mild constitutional varus and (b) the same knee following degenerative cartilage loss in the medial joint space. In this case, joint space narrowing increases the mHKA varus while maintaining the MPTA and LDFA.

Discussions regarding the role of the constitutional alignment of the knee in surgical planning have recently been renewed since publication of a 2021 study by MacDessi et al. that defined nine knee phenotypes in the coronal plane (Figure 1) [12]. This simplified coronal plane alignment of the knee (CPAK) classification was based on two variables: the arithmetic hip‐knee‐ankle angle (aHKA), which described constitutional limb alignment and joint line obliquity (JLO). JLO extends the coronal phenotype classification of a knee from being based on a 3‐class categorisation based on aHKA alone (varus, neutral or valgus) to a 3 x 3 two‐factor classification, which additionally considers the joint line inclination (where JLO values are classified as apex distal, neutral or apex proximal) [7, 9, 12, 18].

The measurements to determine the aHKA and JLO in the MacDessi et al. study were taken on long leg radiographs (LLRs), a technique which is prone to variability due to variations from patient to patient in leg rotation, knee flexion and height of the x‐ray beam [14, 16, 21]. When comparing data from long leg radiography to navigation, the difference between preoperative measurements of mechanical hip‐knee‐ankle angle (mHKA) using the two techniques can be as much as 12° (mean of 4.7°) [21]. In addition, institutions using surgical navigation may not routinely use LLRs in the preoperative process. For both these reasons, it may be desirable to evaluate the CPAK classification using measurements made using surgical navigation technology instead of LLRs. However, given that navigation‐based measurements of LDFA and MPTA do not directly account for cartilage degradation, the CPAK class distributions could differ based on the type of measurement used.

The methodology used in determining the aHKA and JLO is important as these measurements are calculated using anatomical landmarks, namely the medial proximal tibial angle (MPTA) and the mechanical lateral distal femoral angle (LDFA). MacDessi et al. described the constitutional alignment of the lower limb using the formula: aHKA = MPTA–LDFA, where MPTA and LDFA were measured on LLRs. This method of determining alignment estimates the premorbid constitutional alignment and, since it relies solely on bony structures, is insensitive to the effect of joint space narrowing due to wear and tibiofemoral subluxation (Figure 1) [11, 12]. JLO is calculated by adding MPTA and LDFA. When the sum of these two angles exceeds 183°, it indicates an apex proximal joint line, while a sum less than 177° suggests an apex distal joint line.

**Figure 2:**
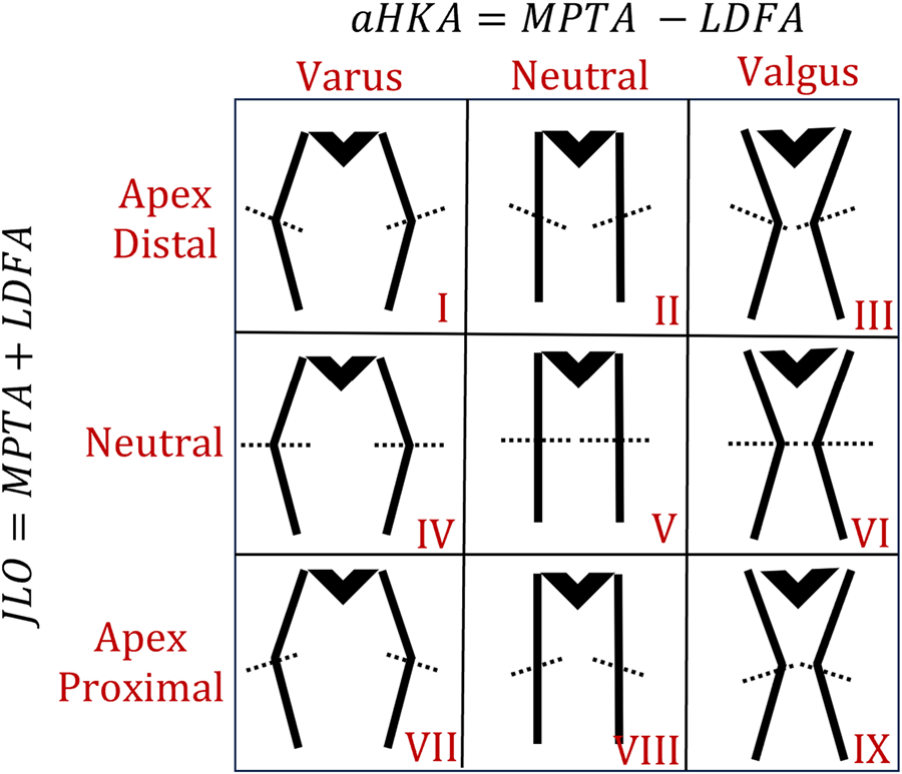
Coronal plane alignment of the knee classification (CPAK) showing the nine knee types. The [12] boundaries defining neutral aHKA and JLO are 0° ± 2° and 180° ± 3°, respectively, as reported in [12]. aHKA, arithmetic hip‐knee‐ankle angle; JLO, joint line obliquity; LDFA, lateral distal femoral angle; MPTA, medial proximal tibial ankle.

In theory, the CPAK classification can be used to guide different alignment strategies in total knee arthroplasty (TKA). Surgeons can choose to perform resections aimed at producing specific postoperative CPAK types. For example, a mechanical alignment strategy should result in a Type 5 knee following surgery (neutral aHKA and neutral JLO), whereas an anatomic alignment strategy should result in a Type II knee (neutral aHKA and apex distal JLO). A kinematic alignment strategy will generally aim to preserve the CPAK type pre to postoperatively. While these alignment strategies aim to produce particular postoperative CPAK classifications, actual postoperative angles used for the classification are rarely measured or reported postoperatively (Figure 2).

Using surgical navigation techniques to determine the MPTA and LDFA in a different population of patients undergoing TKA could result in different CPAK classification distributions compared to those based on LLRs. Given the above, we asked: (1) Does applying the CPAK classification system to measurements obtained from a surgical navigation system result in different class distributions when compared to those reported in [13] and (2) What is the ability of a navigation‐based surgical technique to consistently deliver CPAK Type V when applying a mechanical alignment strategy?

## METHODS

### Study design

**Figure 3:**
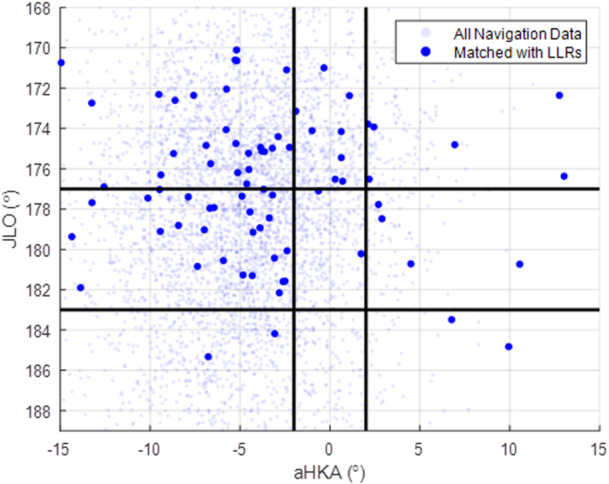
Full dataset of Coronal Plane of the Knee (CPAK) classes acquired using navigated TKA (light blue) and those with matched long‐leg radiographs (LLR) (dark blue). aHKA, arithmetic hip‐knee‐ankle angle; JLO, joint line obliquity.

We analysed data from 4087 TKA procedures undertaken using surgical navigation (OrthoPilot® BBraun Tuttlingen) at our institution between March 2007 and October 2022, with measurements of both the pre and postoperative MPTA and LDFA (Figure 3). The LDFA and MPTA were defined in accordance with [13] but measured using a surgical navigation system. Of these patients, 80 were found to have preoperative LLRs (including cases from 3 different fellowship‐trained surgeons), from which the MPTA and LDFA were measured in accordance with [13] by two clinicians (one fellowship‐trained surgeon and a medical student). A more detailed description of how the MPTA and LDFA are acquired intraoperatively when using the navigation system is provided in the following sections.

### Surgical technique and measurement

**Figure 4:**
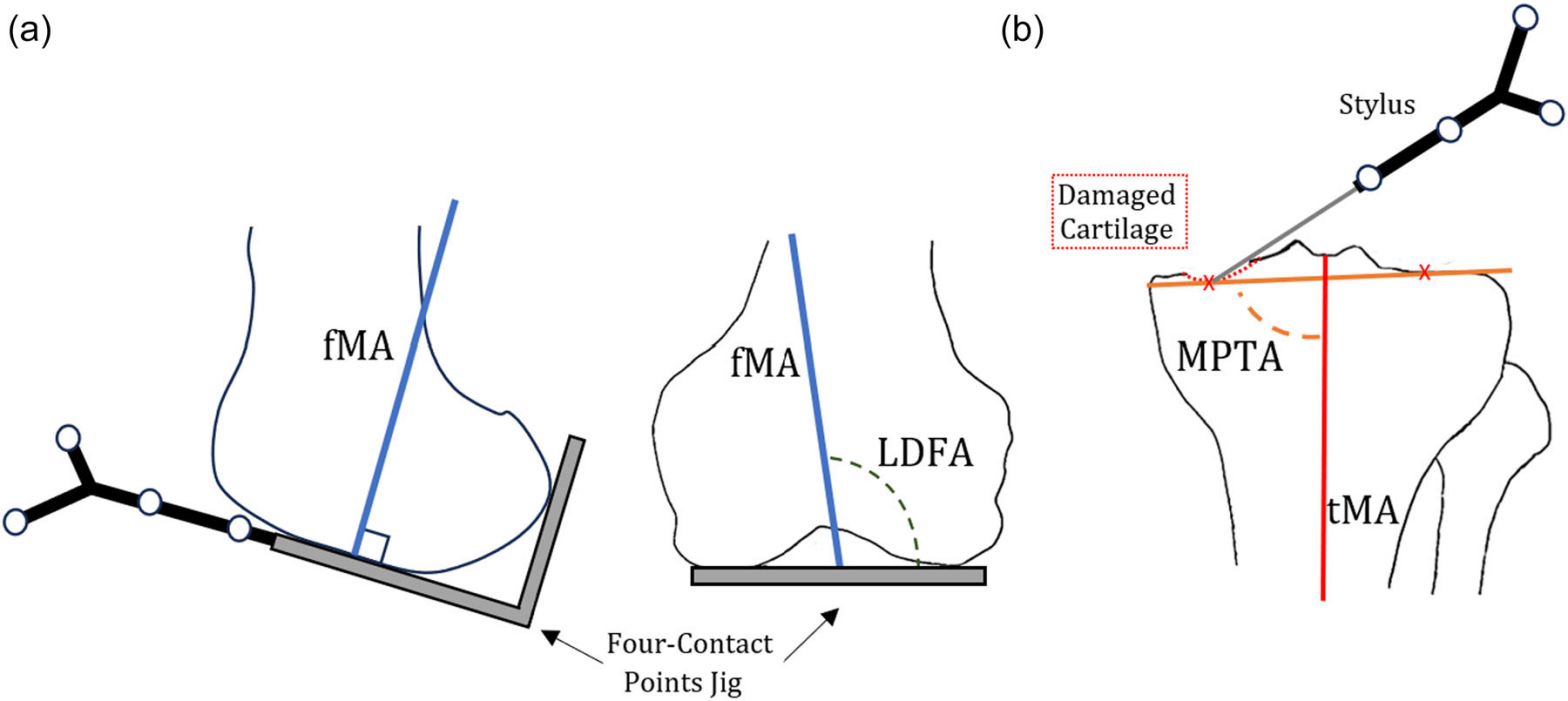
During intraoperative registration, surgeons used precalibrated tools to collect relevant landmarks on the knee to calculate the lateral distal femoral angle (LDFA) and the medial proximal tibial angle (MPTA). (a) The LDFA was measured using an instrument called the ‘four‐contact points jig’, a tool which was optically tracked and positioned perpendicular to the femoral mechanical axis (fMA) in the sagittal plane. (b) The MPTA was measured by capturing the lowest point of the most damaged tibial plateau and the highest point of the less damaged tibial plateau to calculate the joint line relative to the tibial mechanical axis (tMA). Additionally, we opted not to make any further corrections to the MPTA or LDFA to account for cartilage loss.

During intraoperative registration, the surgeons collected kinematic and anatomical landmarks to build the virtual model used to plan and execute the TKA. Following optical tracker fixation above and below the knee joint (i.e., in the femur and in the tibia), the surgeons followed the standard data registration, including kinematic acquisition data of the hip and the knee according to recommendation and acquisition of relevant landmarks using a precalibrated pointer (stylus) (Figure 4) [19]. Landmarks on the knee were sequentially collected on the femur, followed by the tibia. To calculate the LDFA, surgeons recorded the most distal and prominent points of the medial and lateral condyles of the femur using a special instrument called the ‘four‐contact points jig’ (OrthoPilot® BBraun Tuttlingen) [19]. This instrument was optically tracked and rested on the cartilage surface of the distal and posterior femur; measurements were taken when this tool was reported to be nominally perpendicular to the lateral projection of the mechanical axis (i.e., the line connecting the centre of the hip and centre of the knee when viewed in the mediolateral direction). For the MPTA, surgeons used the pointer to record the lowest point of the most damaged tibial plateau and the highest point of the less damaged tibial plateau, as well as the centre of the knee; these points were used to calculate the joint line and tibial mechanical axis. The MPTA was defined to be the angle between the tangent of the upper tibia landmarks and the tibial mechanical axis. At the end of the registration, the mechanical axis alignment approach specifies that the desired cuts are to be made perpendicular to both the tibial mechanical axis (tMA) and femoral mechanical axis (fMA).

### CPAK classification

The CPAK classification is based on two distinct coronal plane measurements of the knee: the aHKA and JLO (Figure 3). We calculated the aHKA as the difference between the MPTA and the LDFA and the JLO as the sum of the MPTA and LDFA, as described in [13]. An aHKA of less than − 2° indicates constitutional varus alignment, while an aHKA of more than + 2° indicates constitutional valgus limb alignment. A JLO of more than 183° indicates an apex proximal joint line, whereas a JLO of less than 177° indicates apex distal. The postoperative aHKA was computed as the difference between the measured coronal resection angles on the proximal tibia and distal femur relative to the mechanical axis recorded by the navigation system. Similarly, the postoperative JLO was calculated as the sum of these angles.

### Statistical analysis

Interrater reliability for MPTA and LDFA measured on LLRs between the two clinicians was assessed using the intraclass correlation coefficient (ICC; two‐way mixed effects, absolute agreement, single rater). Scatterplots were created to demonstrate alignment distributions for the various arthritic groups. Fisher′s Exact test was used to compare differences in CPAK class frequencies and a *F*‐test to compare the variance of lower limb alignment variable measurements. Statistical significance was set at a *p* < 0.05, and a Bonferroni correction for multiple comparisons was applied. Additionally, a post hoc power analysis was conducted to detect a difference of 2° in aHKA and JLO in the matched cohort of patients with an alpha = 0.05.

### Ethical approval

This study was approved by the Golden Jubilee National Hospital Department of Clinical Governance (#1969). When applying for Health Research Authority (HRA) ethical approval, the authors were informed that the research activity comes under an exemption as per section C6, as the study was limited to retrospective intraoperative data with no patient‐identifying information accessed or used in the study, and that HRA approval was not required.

## RESULTS

The interrater reliability for MPTA and LDFA measured on LLRs between the two clinicians were 0.98 and 0.90, respectively, indicating excellent agreement. The mean signed differences between preoperative MPTA and LDFA values measured using surgical navigation, and LLRs were 0.2° (*σ* = 2.8°, *p* = 0.46) and 1.4° (*σ* = 2.6°, *p* < 0.01), respectively (Table 1). The mean signed differences in preoperative aHKA and JLO were calculated to be −1.7° (*σ* = 3.7°, *p* < 0.01) and 11.2° (*σ* = 3.8°, *p* < 0.01). A post hoc power analysis, based on the primary comparison between preoperative surgical navigation and LLR measurements for aHKA and JLO (*n *= 80), demonstrated that the study achieved a power greater than 0.98 to detect a clinically significant difference of 2° (assuming the observed standard deviation of 3.7°–3.8°).

**Table 1:** Preoperative differences in lower limb alignment variables between surgical navigation a long‐leg radiographs.

Alignment variable	Long‐leg radiographs	Navigation	Difference
MPTA	86.3° ± 3.2°	86.5° ± 3.6°	0.2° ± 2.8°
LDFA	88.8° ± 2.7°	90.2° ± 3.4°	1.4° ± 2.6°*
JLO	175.1° ± 3.3°	176.7° ± 3.7°	1.2° ± 3.8°*
aHKA	−2.4° ± 4.9°	−3.7° ± 5.9°	−1.7° ± 3.7°*

Abbreviations: aHKA, arithmetic hip‐knee‐ankle angle; JLO, joint line obliquity; LDFA, lateral distal femoral angle; MPTA, medial proximal tibial ankle.

* Indicates *p* < 0.05 post‐Bonferroni correction.

**Figure 5:**
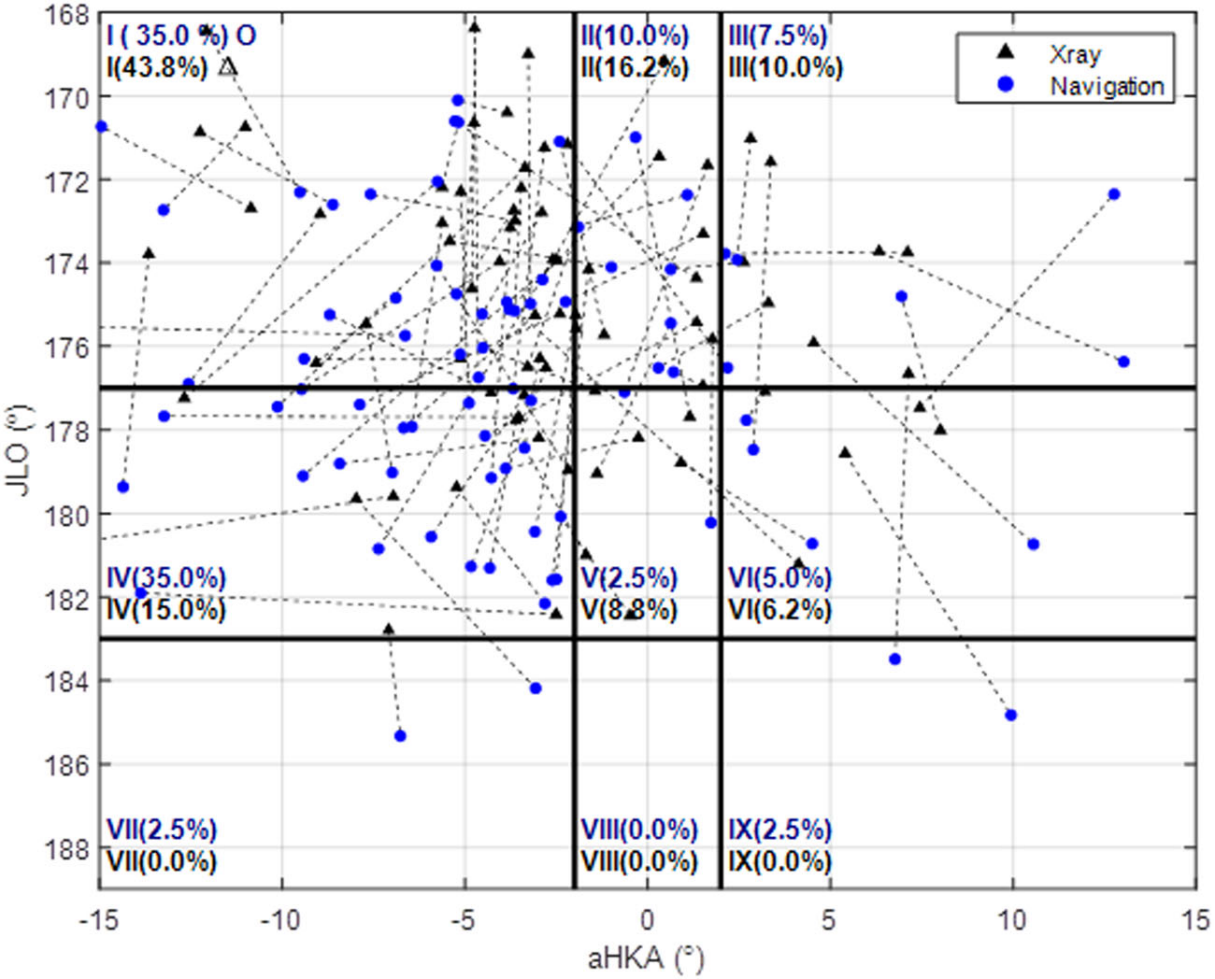
Comparison of Ccoronal plane of the knee (CPAK) class distributions acquired using navigated TKA (blue) and those reported in [12] using long‐leg radiographs (LLRs, black). The dotted lines connect LLR and navigated CPAK measurements of the same patient. The proportion of cases in each class is reported as a percentage. No differences in class assignment frequencies were determined to be statistically significant following the post hoc Bonferroni correction. aHKA, arithmetic hip‐knee‐ankle angle; JLO, joint line obliquity.

Overall, the CPAK class frequency distributions using surgical navigation were broadly similar given that no statistical differences were detected between navigation‐based and surgical navigation‐based measurements (Figures 4 and 5). Most differences in classification frequencies were less than 5%, though the difference was as large as 20% for Type IV—see Figure 5).

The most common CPAK types measured with surgical navigation and LLRs were Type I (varus aHKA, apex distal JLO; 35.0% [*n* = 28] compared to 43.8% [*n* = 35]), Type IV (varus aHKA, neutral JLO; 35.0% [*n* = 28] vs. 15.0% [*n* = 12]) and Type II (neutral aHKA, apex distal JLO; 10.0% [*n* = 8) vs. 16.2% [*n* = 13]). CPAK Types VII, VIII and IX were rare. Despite these similarities in classification frequencies, of the 80 patients included in this analysis, CPAK class assignment was only preserved in 38.8% (*n* = 31) of patients when comparing surgical navigation and LLRs, which indicates that individual patients would frequently be classified differently depending on the measurement technique used.

Consonant with these broad similarities in classification frequencies, the distributions of the LDFA and aHKA measured using the surgical navigation group exhibited variabilities that were not statistically different from measurements made on LLRs (LDFA: *σ* = 3.4° vs. *σ* = 2.7°, *p* = 0.06 and aHKA: *σ* = 5.9° vs. *σ* = 3.7°, *p* = 0.12).

**Figure 6:**
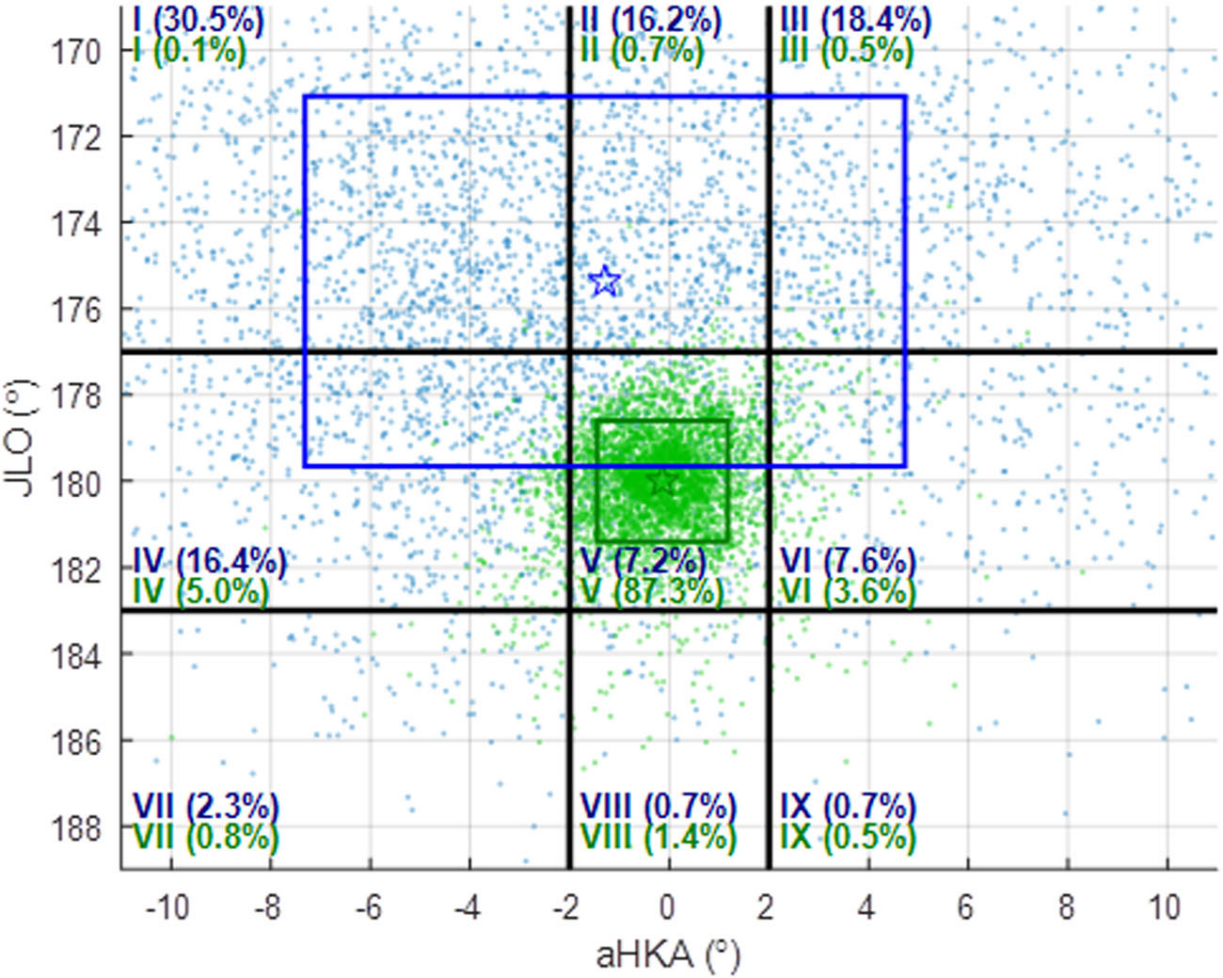
Comparison of preoperative (blue) and postoperative (green) CPAK classifications for cases at our institution, as measured using surgical navigation. The coloured rectangles represent ± one standard deviation from the mean (star). The proportion of cases in each class is reported as a percentage. aHKA, arithmetic hip‐knee‐ankle angle; JLO, joint line obliquity.

Postoperatively, the vast majority of cases resulted in a traditional CPAK classification of Type V; 87.3% (*n* = 3568) when measured using the navigation system (Figure 6). If we extended the acceptance zone for defining neutral aHKA based on the commonly accepted guideline of ± 3° [15], a Type V result would occur in 93.9% of cases. A relatively small number of postop knees were classified as other types. Overall, the percentage of all knees placed with an aHKA of ± 3° (Types II, V and VIII) was 97.0%.

## DISCUSSION

Using statistically indistinguishable from measurements made via LLRs, these group‐level similarities mask a profound individual‐level discordance where class assignment was preserved in only 38.8% of patients. This discrepancy indicates that individual phenotype assignment is highly sensitive to the measurement modality employed, likely due to differences in how navigation and LLRs account for cartilage loss and rotational deformities. Conversely, our findings provide robust, large‐scale validation of the precision of navigation‐assisted mechanical alignment, which successfully delivered the targeted Type V classification in 87.3% of 4087 procedures. Ultimately, these results confirm the reliability of computer‐assisted navigation in executing planned mechanical goals while highlighting the clinical necessity for caution when applying phenotype‐specific strategies across different measurement technologies. Differences in lower‐limb alignment variables and associated CPAK types may arise due to differences in how the LDFA and MPTA are measured using navigation versus LLRs, since the two techniques use somewhat different measurements and anatomical landmarks to assign coordinate systems and measure alignment.

**Figure 7:**
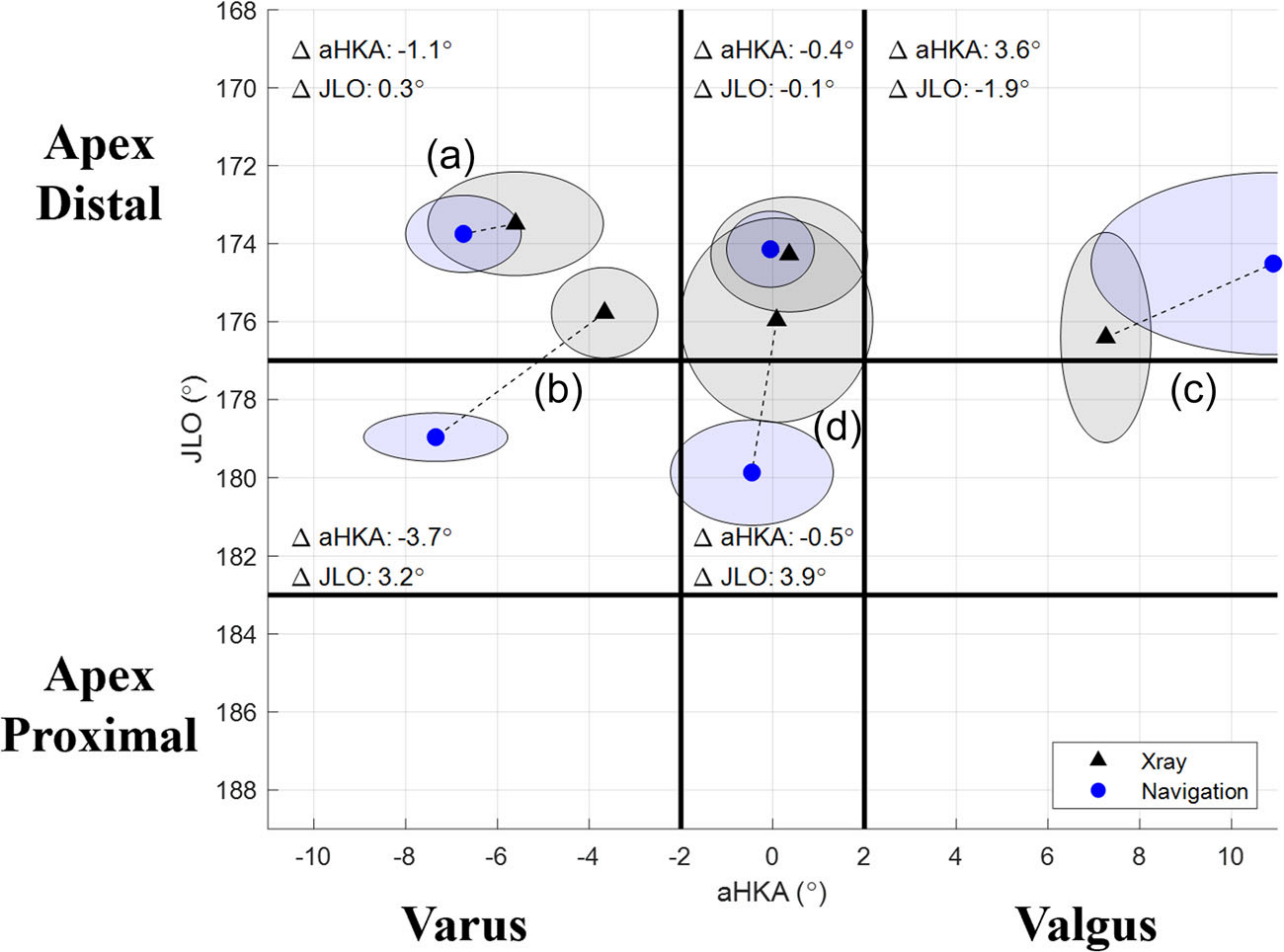
Comparison of mean difference in aHKA and JLO between surgical navigation (blue circle) and LLRs (black triangle), stratified by CPAK Type. The shaded ellipses indicate the standard error of the mean. Labels a–d are used to easier identify connections between LLR CPAK types and their associated surgical navigation measurements. aHKA, arithmetic hip‐knee‐ankle angle; CPAK, coronal plane alignment of the knee; JLO, joint line obliquity; LLR, long leg radiograph.

Measurements of the constitutional alignment of the knee using surgical navigation is affected by cartilage loss and osteophytes, as the bone surface is not directly palpated, so variations in the cartilage thickness across patients contributes to variability (possibly reflected in the nominally wider variance in our measurements of LDFA (*σ* = 3.8° vs. *σ* = 2.1°) and MPTA (*σ* = 3.2° vs. *σ* = 2.1°) using surgical navigation compared with using LLRs). In fact, anticipated patterns in how aHKA and JLO measurements might differ between LLRs and surgical navigation were apparent when patients were stratified by CPAK type (Figure 7). In varus knees, we expect the wear to occur on the medial proximal tibia, lowering the MPTA compared to LLRs and shifting the aHKA towards varus and apex proximal (Figures 7a,b). Similarly, an increase in LDFA in varus knees due to cartilage loss could counteract the MPTA shift, making the JLO shift more towards the horizontal (180°). However, this is less apparent in the Type I knees (Figure 7a) compared to Type IV (Figure 7b). In contrast, the opposite effect is observed in valgus knees, resulting in a shift towards more being valgus and apex distal (Figure 7c).

In a well‐aligned knee (Types II, V and VIII), rotational errors resulting from the 2D projection of LLRs tend to be less pronounced compared to other CPAK types. Therefore, in these neutral CPAK types, the main difference between LLR measurements and navigation lies in the cartilage layer, possibly explaining why only the joint line orientation shifts towards the apex proximal, while overall alignment remains unchanged (Figure 7d). Overall, these factors likely result in some unmodeled shifts in MPTA and LDFA in patients with excessive cartilage wear when measured using a navigation system. Conversely, measurements of CPAK variables based on LLRs also tend to underestimate the degree of varus in the coronal plane in cases with fixed‐flexion contracture in the knee and other rotational deformities, effects which are captured during surgical navigation [5].

Perhaps not too surprisingly, compared to CPAK distributions reported in MacDessi et al., CPAK classes measured using LLRs in this study showed modestly to moderately different proportions of knees in the most common CPAK types. For example, MacDessi et al. reported Type II (32.3%), Type I (19.4%) and Type III (15.4%) as the most common classifications compared to our study′s frequencies of Type I (43.8%), Type II (16.2%) and Type III (10%). The most likely reason for these differences is that this study was conducted on a clinical population comprised of patients from a different part of the world (Scotland, UK) compared to those reported in MacDessi et al.(Australia) [13], and it is likely that there may be moderate differences in constitutional alignment between these populations. For example, the mean age of our cohort was 68 years (range: [45–84], *n* = 80), slightly greater than that reported in MacDessi (mean: 66 years, range: [44–88], *n* = 310), which potentially influenced the distribution of knee types. However, others have reported that the accuracy of measuring aHKA is affected relatively little by age or early osteoarthritis changes [12]. So other demographic differences are likely more relevant here.

This study has several key limitations. CT‐free navigation has several known limitations related to data registration [8]. Landmark data collection relies on the surgeon′s ability to register relevant landmarks accurately and precisely, and there is further variability related to the depth of the cartilage under the pointer on the tibial side. However, overall, navigation systems have proven to be reliable [10], using standardised methods for landmarking [19], and the surgeons who used the system in the cases reported here are high‐volume surgeons with extensive experience using navigation, which reduces the risk of inaccuracy.

Additionally, while our total database was large (*n *= 4087), the matched cohort used for the primary comparison between navigation and LLRs was limited to 80 patients. Although our post hoc power analysis indicated this was sufficient to detect clinically significant differences of 2°, a larger matched cohort might have better captured the distribution of rarer CPAK types, such as VII, VIII and IX, which were nearly absent in our sample.

A critical distinction in this study is the fundamental difference in how computer navigation and standing LLRs handle cartilage and joint loading. Navigation provides a non‐weight‐bearing intraoperative assessment based on direct probing of surface anatomy, whereas LLRs reflect the arthritic joint under weight‐bearing conditions. We deliberately opted not to use universal 2 mm cartilage correction algorithms, as these can introduce unnatural shifts and discontinuities in the CPAK distribution.

This choice likely explains why the individual CPAK class assignment was preserved in only 38.8% of patients; our navigation‐based values reflect the arthritic state at the time of surgery rather than the premorbid state estimated by LLRs. This methodology is supported by Willcox et al. [20], who found that intraoperative navigation measurements do not necessarily correlate with standing LLRs in deformed knees, and Barbotte et al. [1], who noted that while LLR remains the clinical reference, navigation provides unique intraoperative precision. If corrections are desired in the future, we recommend ‘warping’ the distributions of MPTA and LDFA more smoothly to better preserve the underlying patient ordering without introducing artificial discontinuities

We found three other recent studies which applied the CPAK classification to aHKA and JLO parameters acquired though technology‐assisted means rather than using LLRs. Similar to our results, two studies found a higher percentage of knees were classified as Type I (varus and apex proximal, 33.3% [17] and 30% [2, 4], compared with 43.8% for ours) compared to what was reported in MacDessi et al. (19.4%) [13]. However, Clark et al. [2, 4] had a much larger percentage of types classified as Type II (41%) compared to those in our study (16.2% for LLR and 10.0% for navigation), in Orsi et al. (25%) [17], in Edelstein et al. (29.5%) [6] and in MacDessi et al. (32.2%) [13]. Each of these studies applied a correction factor to account for cartilage loss in arthritic knees when calculating preoperative MPTA and LDFA. However, Edelstein et al. created their own correction algorithm to account for joint space narrowing. In their study, the authors iterated through different bounds for mHKA, which constitute a varus or valgus knee and then adjusted the amount of correction to apply to account for cartilage loss in those cases. The authors then selected the combination of these parameters which minimised the root mean square error relative to LLRs for MPTA and LDFA [6].

In our study of postoperative CPAK classifications, we used a mechanical alignment goal for the surgeries rather than a kinematic alignment approach. However, in future studies, we intend to compare clinical outcomes between patients who maintained their preoperative CPAK type postoperatively (similar to a kinematic alignment strategy) and those whose CPAK type was changed. It is also worth mentioning that the CPAK classification, regardless of the measurement tool used, only assesses coronal knee alignment, whereas a full plan for TKA also involves considerations related to sagittal and transverse plane geometry, so the factors captured in the CPAK classification are, on their own, insufficient to fully guide all surgical decisions [3].

## CONCLUSION

In this study, using surgical navigation to evaluate the preoperative CPAK classification rather than LLRs resulted in broadly similar distributions of CPAK types relative to one another (no statistically significant differences), as well as relative to the distributions in the original study that defined the CPAK classification. However, at an individual level, the CPAK class assignment was preserved in only 38.8% of patients, indicating that the measurement technique frequently alters how a specific patient is classified. Additionally, when applying a mechanical alignment strategy, the navigation system successfully delivered a Type V classification in 87.3% of cases. This confirms the reliability of computer‐assisted navigation to accurately and precisely guide mechanical alignment targets.

## AUTHOR CONTRIBUTIONS

All authors contributed to the study conception and design. Material preparation, data collection and analysis were performed by Matthew D. Hickey, Alexander Tham and Meraj Akhtar. Supervision was provided by Antony J. Hodgson, Alistair Ewen and Frederic Picard. The first draft of the manuscript was written by Matthew D. Hickey and all authors commented on previous versions of the manuscript. All authors read and approved the final manuscript.

## CONFLICT OF INTEREST STATEMENT

One of the authors (Antony J. Hodgson) certifies that they hold shares in Trauma Surgical Systems and several patents that are broadly relevant to the work. One of the (Frederic Picard) certifies that they hold shares in Surgiconcept Ltd. and Prometheus Regeneration R&D Ltd. and several patents that are broadly relevant to the work.

## ETHICS STATEMENT

This study was approved by the Golden Jubilee National Hospital Clinical Governance Committee (#1969). When applying for Health Research Authority (HRA) ethical approval, the authors were informed that the research activity comes under an exemption as per section C6 as the study was limited to retrospective intraoperative data and no personal patient data was accessed or used in the study, and that HRA approval was not required.

## Data Availability

Research data are not shared.
